# Prevention and treatment of cardiovascular disease in Ethiopia: a cost-effectiveness analysis

**DOI:** 10.1186/s12962-016-0059-y

**Published:** 2016-08-12

**Authors:** Mieraf Taddesse Tolla, Ole Frithjof Norheim, Solomon Tessema Memirie, Senbeta Guteta Abdisa, Awel Ababulgu, Degu Jerene, Melanie Bertram, Kirsten Strand, Stéphane Verguet, Kjell Arne Johansson

**Affiliations:** 1 Department of Global Public Health and Primary Health Care, University of Bergen, Bergen, Norway; 2 Department of Internal Medicine, School of Medicine, Addis Ababa University, Addis Ababa, Ethiopia; 3 Federal Ministry of Health (FMOH), Addis Ababa, Ethiopia; 4 Management Science for Health, Addis Ababa, Ethiopia; 5 World Health Organization, Geneva, Switzerland; 6 Department of Global Health and Population, Harvard T.H. Chan School of Public Health, Boston, MA USA

**Keywords:** Cost-effectiveness analysis, Cardiovascular disease, Ischemic heart disease, Stroke, Prevention, Treatment, Ethiopia

## Abstract

**Background:**

The coverage of prevention and treatment strategies for ischemic heart disease and stroke is very low in Ethiopia. In view of Ethiopia’s meager healthcare budget, it is important to identify the most cost-effective interventions for further scale-up. This paper’s objective is to assess cost-effectiveness of prevention and treatment of ischemic heart disease (IHD) and stroke in an Ethiopian setting.

**Methods:**

Fifteen single interventions and sixteen intervention packages were assessed from a healthcare provider perspective. The World Health Organization’s Choosing Interventions that are Cost-Effective model for cardiovascular disease was updated with available country-specific inputs, including demography, mortality and price of traded and non-traded goods. Costs and health benefits were discounted at 3 % per year. Incremental cost-effectiveness ratios are reported in US$ per disability adjusted life year (DALY) averted. Sensitivity analysis was undertaken to assess robustness of our results.

**Results:**

Combination drug treatment for individuals having >35 % absolute risk of a CVD event in the next 10 years is the most cost-effective intervention. This intervention costs US$67 per DALY averted and about US$7 million annually. Treatment of acute myocardial infarction (AMI) (costing US$1000–US$7530 per DALY averted) and secondary prevention of IHD and stroke (costing US$1060–US$10,340 per DALY averted) become more efficient when delivered in integrated packages. At an annual willingness-to-pay (WTP) level of about US$3 million, a package consisting of aspirin, streptokinase, ACE-inhibitor and beta-blocker for AMI has the highest probability of being most cost-effective, whereas as WTP increases to > US$7 million, combination drug treatment to individuals having >35 % absolute risk stands out as the most cost-effective strategy. Cost-effectiveness ratios were relatively more sensitive to halving the effectiveness estimates as compared with doubling the price of drugs and laboratory tests.

**Conclusions:**

In Ethiopia, the escalating burden of CVD and its risk factors warrants timely action. We have demonstrated that selected CVD intervention packages could be scaled up at a modest budget increase. The level of willingness-to-pay has important implications for interventions’ probability of being cost-effective. The study provides valuable evidence for setting priorities in an essential healthcare package for CVD in Ethiopia.

## Background

Cardiovascular disease (CVD) is the leading cause of mortality globally. The Global Burden of Disease study estimated that about 32 % of all deaths worldwide in 2013 were caused by CVD [1], with about 80 % of these deaths occurring in low-and middle-income countries (LMIC) [1, 2].

Approximately 9 % of all deaths in Ethiopia in 2012 were caused by CVD according to World Health Organization (WHO) estimates [3]. Small-scale local studies also reported an increasing burden from CVD and its risk factors, especially in urban settings in Ethiopia [4–15]. In a systematic review of studies conducted in Ethiopia between 1960 and 2011, CVD was reported to be among: (a) the prevalent causes of morbidity (range 4–24 %); (b) the main causes of hospital admission, especially among those older than 60 years (range 3–31 %); (c) the leading causes of medical intensive care unit admission (range 8.9–9.8 %); and (d) among the major causes of mortality (range 6.5–24 %) [15]. In Ethiopia’s capital, Addis Ababa, an estimated 25 % of all household deaths between 2006 and 2009 and 11 % of all hospital deaths between 2002 and 2010 were attributed to CVD [7, 8]. Myocardial infarction, stroke and hypertensive heart disease accounted for about 75 % of CVD deaths [7, 8]. Modifiable risk factors like smoking, high cholesterol and high blood pressure explain the major share of the CVD burden [16, 17]. The prevalence of hypertension in Ethiopia is estimated to range from 16 to 30 % [5, 6, 13, 14].

WHO recommends a combination of population-wide and individual-based prevention and basic treatment strategies for successful control of CVD [18, 19]. Current coverage of such interventions is low in Ethiopia. Only about a quarter of the patients diagnosed with CVD at two referral hospitals in Ethiopia were found to be on medication [6, 9].

Cognizant of the increasing burden from non-communicable diseases (NCDs), the Federal Ministry of Health of Ethiopia (FMOH) has launched a National Strategic Action Plan (NSAP) for Prevention and Control of NCDs, envisioning the scale-up of an essential package of NCD interventions targeting the four major NCDs, including CVD [20]. With Ethiopia’s meager health spending of only about US$ 21 per capita per year in 2011 [21], it is imperative to identify the most efficient strategies for further scale-up.

Cost-effectiveness analysis is a key tool to assist policy makers in selecting the most efficient strategy among competing alternatives. WHO-CHOICE (Choosing Interventions that are Cost-Effective) has undertaken cost-effectiveness analysis of CVD interventions for the major regions in low- and middle-income countries [22–24]. Regional estimates have limited relevance to country-level decision making due to variation in key parameters. Parameters such as demography, epidemiology, costs and coverage of interventions vary widely across countries within the same regions, warranting the need for local evidence for better decision-making [23, 25–27]. To our knowledge there is no local evidence on cost-effectiveness of CVD interventions in Ethiopia. We therefore intend to fill this knowledge gap and inform the process of evidence-based resource allocation and priority setting for essential package for CVD interventions in Ethiopia.

This paper’s objective is to undertake a cost-effectiveness analysis of primary prevention, acute treatment and secondary prevention of ischemic heart disease (IHD) and stroke in an Ethiopian setting.

## Methods

We performed a generalized cost-effectiveness analysis of prevention and treatment strategies for CVD in an Ethiopian setting based on the WHO-CHOICE approach whereby, cost-effectiveness of each intervention is assessed compared with a ‘no intervention’ scenario [28]. Box 1 below depicts key socio-demographic and economic indicators for Ethiopia. A brief description of the interventions assessed, the modeling approach and the country-specific revisions are outlined below.

**Box 1:** Key socio-demographic and economic parameters for Ethiopia, 2013/14

Parameter	Level	Source
Total population	96.96 million	
Life expectancy at birth	64 years	[29]
GDP per capita	US$505	
Currency exchange rate to US$	17.7	
PPP exchange rate	7.08	
Total health expenditure (annual)	US$1.6 billion	
Per capita spending on health (annual)	US$21	[21]
Number of health facilities		[30]
Hospital	189	
Health center	3547	
Health post	16,251	

### Interventions

Fifteen single interventions and sixteen integrated intervention packages were assessed. Interventions target individuals without a history of established CVD but at risk of developing a CVD event; those with an acute CVD event; and those with a history of established CVD event. Interventions were selected based on the recommendations of WHO and local experts and scientific evidence of effectiveness. Full description of the interventions is outlined in Table 1.

**Table 1:** Description of interventions assessed

Intervention	Description	Health facility stay	Laboratory/imaging
Acute myocardial infarction		9 hospital bed days at tertiary level	CBC, blood glucose, PT, INR, aPTT and serum lipid profile (3 times) plus ECG and RFT twice
Aspirin	Aspirin 325 mg po daily 30 days		
ACE-inhibitor	Enalapril 20 mg po daily for 30 days		
Beta-blocker	Atenolol 50 mg po daily for 28 days		
Aspirin + clopidogrel	Aspirin 325 mg + clopidogrel 300 mg 30 days		
Thrombolytic	Streptokinase 1.5 million i-u		
Primary PCI	Insertion of ballon-tipped catheter with stent into blocked area	6 hospital bed days at tertiary level	
Post-acute myocardial infarction			
Aspirin	ASA 100 mg po daily	4 hospital visit per year (year 1–3) 3 hospital visit per year (year 4–10) at primary hospital	CBC, LFT, RFT, serum lipid profile, serum electrolyte
ACE-inhibitor	Enalapril 20 mg po daily		
Beta-blocker	Atenolol 50 mg po daily		
Statin	Simvastatin 40 mg po daily		
Acute stroke			
Aspirin	Aspirin 160 mg po daily for 1 month	30 hospital bed days at level 3	CBC,PT, INR, aPTT, serum glucose, serum lipid profile, RFT,LFT and serum electrolyte plus brain CT,ECG &CXR once
Post-acute stroke			
Aspirin	Aspirin 100 mg po daily	4 hospital visit per year (year 1–3) 3 hospital visit per year (year 4–10) at primary hospital	CBC, RFT, LFT, serum lipid profile, serum electrolyte
ACE-inhibitor	Enalapril 20 mg po daily		
Statin	Simvastatin 40 mg po daily		
Primary prevention of IHD and stroke			
Anti-hypertensive treatment for SBP (>140 or >160 mmHg)	HCT 25 mg + Atenolol 50 mg po daily	4 visit to a health center for the first year followed by 3 visits per year for the remaining 9 years. Additionally, 20 % will have 1.5 visit per year at primary hospital	RFT, serum lipid, blood glucose, U/A
Cholesterol lowering treatment for total cholesterol (>5.7 or >6.2 mmol/l)	Simvastatin 40 mg po daily		LFT, serum lipid, blood glucose, U/A
Combination drug treatment for absolute CVD risk (>5, >15, >25, >35 %)	ASA 100 mg + Hydrochlorothiazide 25 mg + Atenolol 50 mg + Simvastatin 20 mg		RFT, LFT, serum lipid, blood glucose

The intervention packages for ‘acute MI’, ‘post-acute MI’, and ‘post-acute stroke’ were formed as combinations of the drugs under the single interventions during the same health facility stay and the same laboratory investigation requirements as the respective single interventions. A complete list of all the interventions is provided in Table 4

*MI* myocardial infarction; *IHD* ischemic heart disease; *SBP* systolic blood pressure; *CBC* complete blood count; *PT* prothrombin time; *ECG* electrocardiogram; *RFT* renal function test; *LFT* liver function test; *U/A* urinalysis

For primary prevention, individual-based drug regimens based on either the level of systolic blood pressure (SBP), the level of total serum cholesterol or the absolute risk of developing a CVD event over the next 10 years were assessed. Absolute risk is determined based on well-known CVD risk factors (age, gender, SBP, smoking status, body mass index and total serum cholesterol level) [18, 19]. The distribution of mean risk factor levels and smoking status in the population was stratified by age and gender based on the estimates from WHO’s Comparative Risk Assessment project for East Africa region. Estimates of relative risk of developing a CVD event per unit increase in the level of risk factors was then applied to estimate the individual level relative risk of developing a CVD event which is then used to extrapolate the absolute risk of CVD event at population level [19, 31]. The drug regimens are to be delivered on an outpatient basis at health centers and constitute: (a) a beta-blocker and a diuretic at SBP of >140 mmHg or >160 mmHg; (b) statin treatment at serum cholesterol level of >5.7 mmol/l or >6.2 mmol/l; and (c) a combination of aspirin, beta-blocker, diuretic and statin-based on the absolute risk of a CVD event for four thresholds (>5, >15, >25 or >35 %) respectively.

Interventions for acute myocardial infarction (AMI) constitute treatment with aspirin, streptokinase, clopidogrel, beta-blocker, ACE-inhibitor and surgical revascularization with percutaneous coronary intervention (PCI) on an inpatient basis. Aspirin is used for acute treatment of ischemic stroke; and beta-blocker, aspirin, ACE-inhibitor and statin for secondary prevention of IHD and stroke. Interventions were first assessed individually; clinically relevant packages were then formed, building on the intervention with the lowest cost-effectiveness ratio.

Given the current low coverage of interventions—less than 5 %, based on experts’ recommendations—we set modest target coverage of 20 % for all of the interventions.

In the absence of local evidence, efficacy estimates were drawn from previous randomized controlled trials and meta-analyses performed elsewhere (Table 2) [32–46]. Efficacy estimates were adjusted by target coverage and patient adherence level [47–49].

**Table 2:** Effectiveness assumption used in the model expressed in percentage reduction in the outcome of interest

Intervention	Outcome affected	Efficacy in %	Source
Acute myocardial infarction			
Aspirin	28 day mortality	22 (15, 29)	[31, 36]
ACE-inhibitor	28 day mortality	7 (2, 11)	[37, 40]
Beta-blocker	28 day mortality	13 (2, 23)	[37, 40]
Streptokinase	28 day mortality	26 (17, 31)	[36]
ASA + clopidogrel	28 day mortality	32 (17, 47)	[31, 34]
PCI	28 day mortality	61 (38, 75)	[33, 36, 41]
Post-acute myocardial infarction			
Aspirin	Case fatality rate	13 (2, 22)	[31, 66]
ACE-inhibitor	Case fatality rate	23 (14, 30)	[42]
Beta-blocker	Case fatality rate	23 (16, 30)	[43]
Statin	Case fatality rate	19 (15, 24)	[44, 67]
Acute ischemic stroke			
Aspirin	28 day case fatality rate	5 (1, 9)	[31]
Post-acute stroke			
Aspirin	Case fatality rate	16 (2, 29)	[31]
ACE-inhibitor	Case fatality rate	16 (12, 30)	[45]
Statin	Case fatality rate	24 (16, 37)	[35]
Primary prevention of IHD and stroke			
Anti-hypertensive treatment for systolic blood pressure (>140 or >160 mmHg)	Difference between actual systolic blood pressure and 115 mmHg	33 (31, 44)	[40, 46, 68]
Cholesterol lowering treatment for total cholesterol (>5.7 or >6.2 mmol/l)	Serum level of total cholesterol	20 (17, 23)	[27, 44]
Combination drug treatment for absolute risk of CVD (>5, >15, >25, >35 %)	Effect on the level of systolic blood pressure plus serum cholesterol plus aspirin	(33) + (20) + (18)	[27, 40, 44, 46, 66, 68]

### Modeling approach

The WHO-CHOICE’s CVD model for East Africa was used to undertake the analysis [50]. The model was updated with age and sex distribution, birth rate and background mortality rate for Ethiopia [51–53]. In the absence of national data on the current level of incidence, prevalence and mortality rates of IHD and stroke and the distribution of CVD risk factors, the analysis used respective estimates for the East Africa region [22, 23, 31, 50, 52].

The effect of primary prevention interventions is modeled through their impact on the level of risk factors, which is used to recalculate the expected incidence rate for IHD and stroke after implementing the specific intervention. The new incidence rate is applied to estimate the reduction in mortality from the respective diseases. Interventions targeting AMI and acute stroke were modeled through the interventions’ impact on 28-day case fatality rate, while secondary prevention interventions were modeled through their impact on post–acute case fatality rate. The effect of interventions was assumed to be the same across sub-groups.

We used PopMod, a multi-state population model, to estimate the health benefits in disability adjusted life years (DALYs) averted for the Ethiopian population resulting from changes in CVD risk due to specific interventions.

The population in the model is divided into age–sex categories of one-year intervals which are further stratified into four health states: (a) those having IHD; (b) those having stroke; (c) those having both; and (d) those without any of the conditions. Transition between states is dictated by the respective incidence, case fatality and mortality rates. Disability weights for the health states were drawn from the Global Burden of Disease Study 2010 [54]. PopMod traces the changes in population size in each age–sex category over a lifetime of 100 years by standard life table methods with and without specific interventions (‘no intervention’ scenario). Interventions are implemented for 10 years, after which the epidemiologic rates are taken back to the ‘no intervention’ level. Births and background mortality are taken into account [31, 55]. The expected health benefits of the current coverage level of interventions are eliminated to create a hypothetical reference case of null scenario. The model provides removal of the benefits of current coverage of interventions, thereby allowing recalculation of the incidence, prevalence and case fatality rates for MI and stroke, assuming a scenario where the currently implemented interventions are stopped. The health benefits are reported in terms of DALYs averted, discounted at 3 % per year without age weighting. The model has been used to undertake CEAs of various interventions in multiple settings [22]; and details have been published elsewhere [23, 24, 55].

### Costs

A healthcare provider perspective was used for analysis and hence only program costs, training costs and patient-related costs to the provider were taken into account. Program costs constitute the cost of development and administration of an intervention at national and sub-national levels. This includes cost of administration and planning, media and communication, law enforcement, training, monitoring and evaluation. Patient-related costs consist only of direct medical costs incurred by the provider at the point of service delivery, including hospital bed days, outpatient visits, drugs and laboratory [28]. The analysis did not include direct non-medical costs such as transportation and indirect costs to patients and care givers such as lost productivity. The ingredients costing approach was employed whereby the quantities of resources required to deliver the interventions and respective unit prices were accounted for separately (Table 3). The quantities of resources used were largely determined based on WHO-CHOICE assumptions. We updated the prices of relevant laboratory tests and imaging using pricing from two public hospitals in Addis Ababa (Tikur Anbessa teaching hospital and Zewditu hospital). Salary scale of the health workforce was based on the FMOH of Ethiopia. Equipment and material prices were based on WHO price estimates for Ethiopia for the year 2012/13 [56] and drug prices were based on the lowest supplier prices for 2012, as noted in the International Drug Price Indicator Guide [57]. WHO-CHOICE’s transport multiplier factor was applied to the drug prices. The total cost of an intervention was then calculated as the sum of the product of the quantities of resources with their respective unit prices. As recommended by WHO-CHOICE costs were discounted at an annual rate of 3 % [28] and reported in 2012 US$.

**Table 3:** Price of intervention inputs applied in the model in Ethiopian birr 2012

	Unit price		Unit price
Salary scale for human resource			
Medical specialist	112,781	Director of public health	51,293
Medical officer	76,723	Public health specialist	94,712
Nursing director/manager	64,728	Public health assistant	28,339
Registered nurse	28,339	Health educator/trainer	28,339
Health worker	51,293	Social/welfare worker	28,339
Source: Federal Ministry of Health, Ethiopia 2012			
Health facility visit/stay			
Hospital bed days		Health facility visit	
Primary hospital	52.52	Primary hospital visit	18.58
Secondary hospital	54.76	Secondary hospital visit	21.17
Tertiary hospital	70.81	Tertiary hospital visit	22.06
Percutaneous coronary intervention^a^ 63,000		Health center visit	23.00
Source: WHO_CHOICE [69]			
Laboratory and imaging			
Complete blood count	20	Blood glucose	10
Prothrombin time (INR)	15	Urinalysis	5
aPTT	15	Liver function test	30
Serum electrolytes	45	Total cholesterol	7
Renal function test	20	Serum lipids	42
Blood glucose	10	CT scan	600
Echocardiography	150	Endoscopy	400
Source: Tikur Anbesa teaching hospital and Zewditu memorial hospital			
Drugs			
ASA 100 mg	0.08	Simvastatin 20 mg	0.25
Enalapril 10 mg	0.05	Streptokinase 1.5 iu	601.8
Atenolol 50 mg	0.06	Clopidogrel 75 mg	0.55
		Hydrochlorothiazide 25 mg	0.08
Source: International drug price indicator [57]			

^a^Unit price per procedure. The program cost was assumed to be double the program cost required for other acute myocardial infarction interventions

### Cost-effectiveness

All interventions were assessed compared to ‘no intervention’ scenario first, followed by incremental analysis between mutually exclusive interventions. Average cost-effectiveness ratios (ACERs) were estimated dividing the incremental cost by incremental effects of each intervention compared with a ‘no intervention’ scenario. In order to assess the relative cost-effectiveness of mutually exclusive interventions, incremental cost-effectiveness ratios (ICERs) were estimated as the ratio of the incremental cost to incremental effects for moving from one intervention to the next more effective intervention, starting from the null scenario. Interventions that are more costly and less effective than their comparators or those having higher ICER than their more effective comparator are designated as dominated. ACERs and ICERs are reported in US$ per DALY averted for the year 2012.

### Uncertainty analysis

A probabilistic sensitivity analysis was conducted using Monte Carlo League (MCLeague) software to assess the effect of uncertainty surrounding the costs and effectiveness estimates [58]. A truncated normal distribution was used to execute 1000 simulation runs with 15 and 25 % coefficient of variation for costs and effectiveness estimates, respectively. We assessed interventions that were not dominated by respective comparators in each intervention category. In addition, one-way sensitivity analysis was undertaken, applying the lower boundary of the effectiveness range; doubling the price of drugs, procedures and laboratory tests; a zero discounting rate to health benefits; and applying 50 % of the effectiveness point estimates (Tables 1, 2).

## Results

Treatment of acute myocardial infarction with ACE-inhibitor costs the least at US$2.4 million annually. Combination drug treatment to individuals having >5 % absolute risk of developing a CVD event incurs the highest annual cost US$26.9 million— and generates the highest annual health benefit of 190,000 DALYs averted. Treatment of acute stroke with aspirin generates the smallest annual health benefit. The estimated annual costs, health benefits, ACER and ICERs for all interventions are presented in Table 4 below.

**Table 4:** Annual cost, annual health benefits and cost-effectiveness ratio of selected CVD interventions in Ethiopia

Intervention description	Annual cost in million US$	Annual DALYs averted (discounted)	Annual DALYs averted (undiscounted)	ACER	ICER
Acute myocardial infarction					
ACE-inhibitor	2.37	316	422	7531	Dominated
Beta-blocker	2.38	586	784	4057	Dominated
ASA	2.38	990	1325	2200	Dominated
Streptokinase	2.82	1170	1566	2408	Dominated
ASA + clopidogrel	2.38	1441	1927	1556	Dominated
ASA + streptokinase	2.84	2110	2822	1295	Dominated
ASA + streptokinase + ACE-inhibitor	2.85	2396	3205	1149	Dominated
Primary PCI	8.29	2747	3675	3013	Dominated
ASA + streptokinase + ACE-inhibitor + beta-blocker	2.92	2919	3905	999	999
ASA + clopidogrel + PCI	8.5	4015	5370	2115	5087
Acute stroke					
ASA	2.53	63	80	39,892	39,892
Post-acute IHD					
ASA	2.54	245	330	10,345	Dominated
Statin	2.74	310	417	8822	Dominated
Beta-blocker	2.53	488	657	5177	Dominated
ACE-inhibitor	2.55	524	705	4857	Dominated
ASA + beta-blocker	2.57	732	985	3511	Dominated
ASA + beta-blocker + statin	2.82	1038	1397	2717	Dominated
ASA + beta-blocker + statin + ACE-inhibitor	2.88	1557	2096	1849	1849
Post-acute stroke					
ACE-inhibitor	2.87	912	1200	3153	Dominated
ASA	2.86	1013	1348	2821	Dominated
Statin	3.30	1375	1813	2396	Dominated
ASA + statin	3.40	2382	3150	1428	Dominated
ASA + statin + ACE-inhibitor	3.48	3284	4337	1061	1061
Primary prevention of IHD and stroke					
Cholesterol lowering treatment for total chol. >6.2 mmol/l	4.67	8768	15,913	532	Dominated
Cholesterol lowering treatment for total chol. >5.7 mmol/l	10.62	19,073	34,143	557	Dominated
Anti-hypertension treatment for SBP >160 mmHg	7.33	98,880	172,868	74	Dominated
Combination drug treatment for absolute risk of CVD >35 %	7.18	107,687	185,249	67	67
Anti-hypertension treatment for SBP >140 mmHg	19.42	125,712	220,992	154	Dominated
Combination drug treatment for absolute risk of CVD >25 %	9.83	127,957	219,230	77	131
Combination drug treatment for absolute risk of CVD >15 %	14.41	153,877	263,747	94	177
Combination drug treatment for absolute risk of CVD >5 %	26.85	190,391	329,117	141	341

The absolute risk-based approach turns out to be the most cost-effective strategy of all the interventions. Combination drug treatment to individuals having an absolute risk >35 % yields the most value for money with an ICER of US$67 per DALY averted, with ICER reaching US$340 per DALY averted when the risk threshold is lowered to >5 %. When compared with the single risk–factor based approach, the absolute risk-based approach is the most cost-effective option. Notably, initiating treatment at higher CVD risk threshold generates better efficiency gain compared to lower risk thresholds regardless of the approach chosen. This means, for example, that initiating anti-hypertensive drug treatment at SBP of >160 mmHg is more efficient than treatment at >140 mmHg. Of all the interventions for AMI, an integrated package of aspirin, ACE-inhibitors, beta-blockers and streptokinase has the lowest ICER (i.e., US$999 per DALY averted). Provision of interventions in an integrated package generates better efficiency gain and dominates all the single interventions, as shown in Table 4. Moving from the most cost-effective pharmaceutical package to an integrated package that includes the highly skilled intervention PCI, aspirin and clopidogrel raised the ICER substantially—to US$5087 per one additional DALY averted.

Treatment of acute ischemic stroke with aspirin costs US$40,000 per DALY averted. Single drug interventions for secondary prevention of IHD and stroke cost between US$2400 and US$10,300 per DALY averted respectively. Interventions become more efficient when delivered in an integrated package. A package consisting of aspirin, beta-blocker ACE-inhibitor and statin for secondary prevention of IHD costs US$1850 per DALY averted, while a package consisting of aspirin, ACE-inhibitor and statins for secondary prevention of stroke costs US$1060 per DALY averted.

In order to facilitate step-wise selection of the most cost-effective interventions, interventions that dominate their comparators in each category were ranked according to their category-specific ICER. Accordingly, combination drug treatment to individuals having >35 % absolute risk of developing a CVD event is the first intervention to be selected, followed by the same intervention at lower risk thresholds (>25, >15 and >5 %, respectively). A basic integrated package of aspirin, ACE-inhibitor, beta-blocker and streptokinase for AMI and a package of aspirin, statin and ACE-inhibitor for secondary prevention of stroke are the next two interventions that could be selected when more resources become available. Scale-up of combination drug treatment at an absolute risk >35 % to a coverage level of 20 % costs about US$7 million per year and averts 107,000 DALYs annually.

Table 5 presents the results from the one-way sensitivity analysis. At the lower boundary of the effectiveness range, all interventions become less cost-effective. The ACERs increased by a factor of 1.5-to sixfold for AMI and secondary prevention interventions. Primary prevention interventions were less sensitive. Halving the point estimates for effectiveness has a relatively larger impact on the primary prevention interventions, with respective ACERs increasing by a factor of 1.4–1.8. However, even at half point estimate of effectiveness, combination drug treatment to individuals having >35 % CVD risk costs US$94 per DALY averted. Doubling the price of drugs and laboratory tests increases ACERs minimally compared with halving or applying lower limit of effectiveness estimates. All the interventions become more cost-effective at a zero discounting rate for the health benefits (Table 5).

**Table 5:** Average cost-effectiveness ratios for cardiovascular disease interventions under multiple scenarios

Intervention description	Base-case	Undiscounted health benefits^a^	10 % coverage^b^	Double cost^c^	Lower effect^d^	50 % effect^e^
Acute myocardial infarction						
ACE-inhibitor	7526	5626	14,718	7777	26,556	15,172
Beta-blocker	4054	3031	7926	4191	26,556	8171
ASA	2398	1792	4685	2480	3545	4831
Streptokinase	2407	1799	4343	2855	3714	4850
ASA + clopidogrel	1652	1235	3225	1712	2958	3327
ASA + streptokinase	1345	1006	2419	1603	2015	2669
ASA + streptokinase + ACE-inhibitor	1188	888	2133	1411	1903	2342
Primary PCI	3013	2252	4560	4460	4833	5983
ASA + streptokinase + ACE-inhibitor + beta-blocker	998	746	1774	1210	1839	1950
ASA + clopidogrel + PCI	2112	1579	3171	2240	3410	4062
Acute stroke						
ASA	39,896	31,586	75,658	42,135	99,269	79,449
Post-acute myocardial infarction						
ASA	10,345	7701	19,853	11,173	50,593	19,029
Statin	8822	6552	16,139	10,119	10,659	11,594
Beta-blocker	5177	3844	9823	5575	7386	10,296
ACE-inhibitor	4856	3612	9182	5264	6092	6771
ASA + beta-blocker	3512	2610	6612	3835	6556	6793
ASA + beta-blocker + statin	2717	2018	4904	3182	4351	4597
ASA + beta-blocker + statin + ACE-inhibitor	1849	1373	3349	2197	2704	2908
Post-acute stroke						
ACE-inhibitor	3152	2394	5642	3663	3153	3153
ASA	2822	2121	5065	3264	9996	4833
Statin	2397	1820	4046	3042	3427	4355
ASA + statin	1429	1080	2382	1844	2730	2528
ASA + statin + ACE-inhibitor	1061	803	1751	1386	1616	1545
Primary prevention of IHD and stroke						
Cholesterol lowering treatment for total chol. >6.2 mmol/l	532	293	791	738	605	941
Cholesterol lowering treatment for total chol. >5.7 mmol/l	557	311	676	888	636	1002
Anti-hypertension treatment for SBP >160 mmHg	74	42	97	102	77	124
Combination drug treatment for absolute risk of CVD >35 %	67	39	88	103	69	94
Anti-hypertension treatment for SBP >140 mmHg	154	88	172	234	161	263
Combination drug treatment for absolute risk of CVD >25 %	77	45	95	124	80	108
Combination drug treatment for absolute risk of CVD >15 %	94	55	108	157	98	132
Combination drug treatment for absolute risk of CVD >5 %	141	82	153	245	148	199

^a^Undiscounted health benefits

^b^10 % target coverage

^c^Double price for drugs, procedures and laboratory test

^d^Lower boundary of effectiveness estimate

^e^50 % of point estimate of effectiveness

**Fig. 1:**
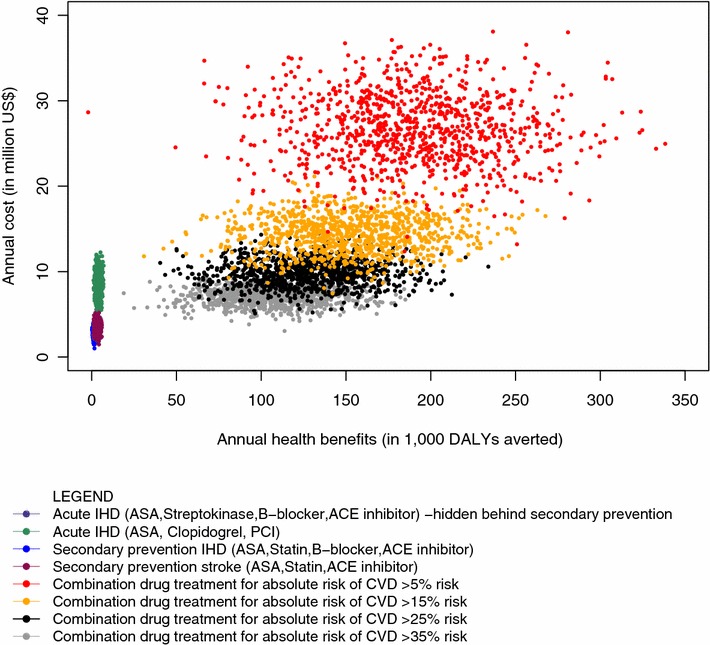
Probabilistic sensitivity analysis of non-dominated CVD interventions in Ethiopia

**Fig. 2:**
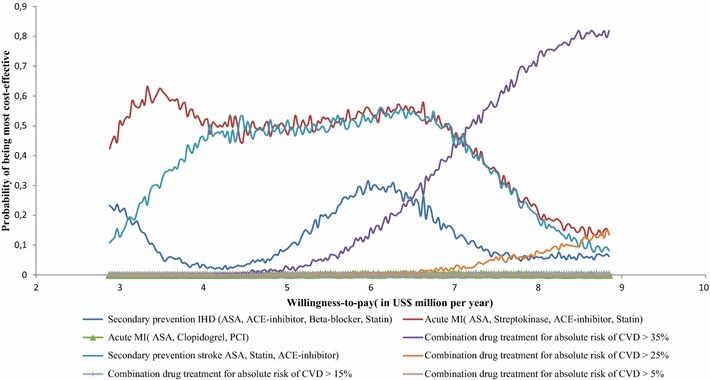
Probabilistic sensitivity analysis of non-dominated CVD interventions in Ethiopia

The probabilistic sensitivity analysis illustrates the serious uncertainty surrounding our results, with wide and overlapping uncertainty ranges for cost and effectiveness estimates (Fig. 1). Budget size has considerable impact on the probability of interventions being cost-effective. At an annual budget of US$3–US$4 million, an integrated package consisting of ASA, streptokinase, ACE-inhibitor and beta-blocker for AMI has the highest probability (0.50) of being the most cost-effective approach. Between US$4 and US$7 million, the probability curve for a secondary prevention package for stroke consisting of aspirin, ACE-inhibitor and statin overlaps on the basic AMI package, making the choice less straight forward. As the budget increases to more than US$7 million per year, combination drug treatment to individuals having more than 35 % absolute risk of CVD stands out as the most cost-effective intervention. However, even at this budget level, the other interventions have less but meaningful probability of being cost-effective (Fig. 2).

## Discussion

Our analysis illustrates that primary prevention of ischemic heart disease and stroke is a more efficient strategy for maximizing population-level health benefits compared with acute treatment and secondary prevention. All primary prevention interventions cost less than US$ 560 per DALY averted. The absolute risk-based approach is more cost-effective than the single risk-factor approaches for primary prevention of CVD. This corresponds with the findings of similar studies for the sub-Saharan Africa region and other regions [23, 24]. The superiority of the absolute risk-based approach is primarily explained by: (a) the linear nature of the correlation between blood pressure and cholesterol level with the risk of CVD event and (b) the tendency for co-existence and interaction between CVD risk factors [19, 59, 60]. The modest ‘efficiency loss’ related to lowering the risk thresholds is due to the larger number of eligible individuals significantly increasing the cost for a relatively modest additional health benefits. It is therefore worthwhile to set the CVD risk threshold at >35 % initially; this can be lowered when more resources become available. The proposed risk threshold of >35 % accords with WHO’s recommendation for resource-limited settings like Ethiopia [18].

All the single pharmacologic interventions for treatment of AMI were dominated by the integrated package consisting of aspirin, ACE-inhibitor, beta-blocker and streptokinase. Delivering interventions in integrated packages yields significant efficiency gain due to savings from program costs and patient costs [23]. This is comparable with the results from the Disease Control Priorities project 2nd edition [61]. Interestingly, the ICER escalates by about six fold if one moves from this basic pharmaceutical package to a highly skilled intervention consisting of PCI, aspirin, and clopidogrel. Although PCI is the treatment of choice for AMI in ideal settings [41, 62], our results indicate the need to prioritize the scale-up of basic pharmacologic regimens for AMI treatment in resource-constrained settings like Ethiopia rather than investing the limited resources on high-standard interventions.

An integrated package of aspirin, beta-blocker, ACE-inhibitor and statin for secondary prevention of IHD and a package of aspirin, ACE-inhibitor and statin for secondary prevention of stroke appears to be the preferred options within their categories. This is in line with the findings of Ortegon et al. for the sub-Saharan Africa region [23]. On the grounds of cost-effectiveness, secondary prevention interventions are ranked lower than primary prevention interventions. This is partly because primary prevention interventions generate a larger population-level aggregate health benefit with relatively lower unit delivery costs [23]. In addition, the need for relatively more frequent follow-up visits at primary hospital level for secondary prevention interventions partly explains higher cost-effectiveness ratios as compared with primary prevention interventions.

Continuing controversy about appropriate thresholds for cost-effectiveness ratios highlights the need for more empirical work in that area [50, 63, 64]. Woods et al. suggested a very low CER threshold of about 50 % of GDP per capita compared with WHO’s recommendation of 1–3 times GDP per capita, which translates to US$505–US$1515 for the year 2013 [50, 63, 64]. Determining the appropriate cost-effectiveness ratio threshold level is beyond the scope of this paper, we therefore discuss the implications of scaling-up the intervention with the lowest ICER and leave the decision to policymakers to further select interventions that best fit the local budget constraint. Accordingly, combination drug treatment to individuals having more than 35 % absolute risk of CVD event is a reasonable starting point. Scale-up of this intervention to a coverage level of 20 % averts 107,000 DALYs annually at a cost of about US$ 7 million per year. This is equivalent to 0.4 % of the 2010/11 annual total health expenditure for Ethiopia [21]. In terms of GDP per capita, the ICER is about 13 % of GDP per capita for 2013.

With the evident escalating burden from CVD and its risk factors [5, 7, 11, 12], investing in primary prevention early on could help Ethiopia partially reduce the need to invest in more costly acute care and secondary prevention measures in the long term. Notably, the most cost-effective combination drug treatment based on an absolute risk approach could be scaled up at the primary health care level, for which Ethiopia has already established a solid foundation [30]. This could facilitate scaling up of the proposed primary prevention interventions at a more modest additional resource requirement than originally estimated. The actual budget implication, however, needs to be assessed separately using appropriate tools.

However, based on the probabilistic sensitivity analysis, the choice of intervention depends on the level of willingness-to-pay. When resources are scarce (<US$7 million annually), a package consisting of aspirin, streptokinase, ACE-inhibitor and beta-blocker for AMI is a preferred option over combination drug treatment for an absolute risk of CVD >35 %, although it ranked lower based on the ICER. It is also worth noting that CEA results are only one of the key parameters to be considered in priority setting. Policy makers need to take into account other important parameters for fair resource allocation, such as severity of disease, equity and financial risk protection [65].

Our study has a number of limitations. We have not included all possible sets of CVD interventions in our analysis. In the absence of country-level data on epidemiology of ischemic heart disease, stroke and the risk factors (incidence, prevalence, and case fatality rate), such estimates were drawn from estimates for the East Africa region. For the same reason, the effectiveness estimates for interventions were drawn from studies in developed settings. This may introduce bias into our cost-effectiveness ratio estimates, as it may be unrealistic to attain the same health benefit level from interventions in an Ethiopian setting; reasons for this may include differences in quality of health services, availability of resources and skilled human resources.

Interventions’ effect is assumed to be uniform across sub-groups with varying risk level. This may have resulted in an overestimation of the potential impact of interventions in individuals with relatively lower risk and underestimation of the potential impact in high risk group. Therefore, detection of the direction of the bias on the final results is not straight forward; our intuition is that the net effect on the final results is very minimal.

PopMod estimates interventions’ health benefits by tracing what would happen to the population with and without the interventions over a lifetime of 100 years. The interventions are assumed to be implemented only for the first 10-year period; the epidemiologic rates are subsequently returned to the ‘no intervention’ level. This only partially captures intervention health benefits; possible extended benefits from interventions on the outcome of interest are missed, resulting in possible underestimation of interventions’ relative cost-effectiveness. Intervention period of more than 10 years involves a high degree of uncertainty and it is difficult to predict how CVD interventions may look like after 10 years from now.

Given the healthcare provider perspective we adopted for the analyses, we have not considered patient and caregiver costs such as transportation and cost of time lost while seeking healthcare. In addition, out-of-pocket expenditure by households constitutes one-third of total health spending in Ethiopia [21]. Such factors might influence households’ decision to access especially prevention strategies that entail repeated visits to health facilities and this aspect requires further exploration.

For primary prevention interventions, we did not consider the cost of screening all eligible individuals to identify ‘at risk’ sub-population groups. Scaling up screening programs could be very costly in low-income settings like Ethiopia [19]; therefore we included the cost of a health center visit and laboratory test only for those identified as ‘at risk’ through opportunistic screening. This would underestimate the potentially huge cost screening could entail at population level.

In addition to the proposed interventions, the potential benefit from sustained life style modification among the public cannot be over-stated for successful prevention and control of CVD in Ethiopia [19].

## Conclusions

In Ethiopia, the escalating burden from CVD and its risk factors warrants timely action. We have demonstrated that selected packages CVD interventions could be scaled up in Ethiopia at a modest budget increase and that combination drug treatment to individuals having more than 35 % absolute risk of CVD event is the most cost-effective intervention. However, the level of willingness-to-pay has important implications for interventions’ probability of being most cost-effective. The study provides valuable evidence for setting priorities in an essential health care package for cardiovascular diseases in Ethiopia.

## Abbrevations

ACE-inhibitor: angiotensin converting enzyme inhibitors
ACER: average cost-effectiveness ratio
AMI: acute myocardial infarction
CBC: complete blood count
CEA: cost-effectiveness analysis
CHOICE: choosing inteventions that are cost-effective
CVD: cardiovascular disease
DALYs: disability-adjusted life years
ECG: electrocardiogram
FMOH: Federal Ministry of Health
GDP: gross domestic product
ICER: incremental cost-effectiveness ratio
IHD: ischemic heart disease
INR: international normalized ratio
LFT: liver function test
LMIC: low- and middle-income countries
NCD: non-communicable disease
NSAP: national strategic action plan
PCI: percutaneous coronary intervention
PT: prothrombin time
RFT: renal function test
SBP: systolic blood pressure
SSA: sub-Saharan Africa
US: United States
WHO: World Health Organization
